# Flood Detection in Steel Tubes Using Guided Wave Energy Leakage

**DOI:** 10.3390/s23031334

**Published:** 2023-01-25

**Authors:** Rito Mijarez

**Affiliations:** Instituto Nacional de Electricidad y Energías limpias, Gerencia de Control, Electrónica y Comunicaciones, Calle Reforma 113, Col. Palmira, Cuernavaca 62490, Mexico; rmijarez@ineel.mx

**Keywords:** flood member detection, piezoelectric transducers, leaky guided waves, time–space between pulses, entropy, digital signal processing

## Abstract

A study that evaluated the use of ultrasonic-guided waves to detect water in hollow pipes is presented. In this work, a guided wave system employed a 40 kHz piezoelectric (PZT) transmitter and a PZT ultrasound transducer. The transmitter was based on a battery-operated microcontroller, and the receiver was composed of a digital signal processor (DSP) module connected to a PC via a USB for monitoring purposes. The transmitter and receiver were attached, non-intrusively without perfect alignment, to the external wall of a steel tube 1 m × 270 mm × 2 mm in size. Flood detection was performed based on guided wave attenuation due to energy leakage from the internal steel wall of the tube to water. Two approaches were carried out. The former was an off-line signal response based on the wavelet energy entropy analysis of a received pulse; the latter was a real-time hit-and-miss analysis centered on measuring the time–space in-between two transmitted pulses. Experiments performed in the laboratory successfully identified flooded tubes.

## 1. Introduction

The health of offshore oil platforms is a critical issue since failures are costly in terms of money, environmental outcomes, and possibly human life. These sub-sea structures are composed of sealed, filled-with-air, hollow steel crossbeams that provide structural support. The exposure of these steel members to salty water causes the penetration of water into tubes due to through-thickness cracks, causing internal flooding in the structure that accelerates their corrosion [1].

Considering that a member can tolerate the occurrence of cracks of through-thickness dimensions, without failing by either fracture or plastic collapse under the offshore maximum loads, by testing for water inside the tubes, the presence of defects can be determined. This issue serves as the basis for a non-destructive testing (NDT) inspection method known as flood member detection (FMD) [2,3]. FMD has long been a maintenance method for offshore oil platforms [4]. Conventionally underwater NDT tools such as ultrasonic probes or gamma radiation sources have been used for flood detection, achieving field-proven results. Nevertheless, these methods are costly to hire regularly and demand special enclosures and severe safety precautions, requiring the deployment of a diver or a remotely operated vehicle (ROV). The implementation of FMD requires a probe handler or frame that should be radial to the component and as low as operationally possible to the steel member. Gamma radiation FMD systems employ narrow beams to reduce unwanted gamma exposure. Generally, these systems possess two arms: one arm sends out a gamma beam, and in the other arm lies the gamma radiation detector. A flooded member exhibits a total gamma-ray reception of much lower value than through air due to energy attenuation [5]. The main downfall is the overexposure of operators to radiation, which could cause short- and long-term health risks [6,7].

Ultrasonic FMD systems generally employ piezoelectric transducers and a pulse-echo technique, in which the analyzed echoes determine the presence of water inside a tube. Time-spaced echoes of ultrasonic pulses reflected from opposing walls and received by the transducer indicate the presence or absence of a flooding tube. Practical applications dictate that probes made of multi-transducers arrays are needed to detect flooding. An operational limitation is that good probe alignment to the wall of the structural member is indispensable to provide reflected echo signals with a good signal-to-noise ratio [8]. Non-ceramic transducers with piezoelectric properties, such as nanostructured thin films (NSTFs), have been reported for FMD to minimize the limitations of piezoceramic materials. An NSTF is a soft structure approximately 50 μm thick that permits superior acoustic coupling to the tube under test. A visual analysis depicted in an oscilloscope exhibited distinct characteristics of the front echo data from a flooded and non-flooded member. The laboratory results displayed a unique signature for a flooded member after the first voltage spike [9]. However, signal processing detection and further testing and trials using different levels of flooding are required to validate this approach. The author proposed the transition of the FMD from a diver-based or ROV-based inspection to a Structural Health Monitoring (SHM) concept for offshore oil platforms under construction [10,11]. The system employs PZT elements, and its electronics were designed to be permanently attached to the inner wall of every tube in the sub-sea structure and powered by normally inert seawater batteries. Upon activation, the sensor transmits ultrasonic encoded information to an online monitoring system, at deck level, to decode and identify the transmitting sensor member. The transmitted signal exploits two communication channels, the former through seawater, the latter using guided waves and the waveguide effect provided by the steel jacket [12,13].

This work presents the initial results obtained from an ultrasonic-guided wave system for the detection of water in hollow steel tubes. The FMD system employs two PZT sensors, one acting as a transmitter exciting 40 kHz guided waves and the other working as a receiver. The transmitter is made of a microcontroller, a signal booster, and a PZT crystal and is powered by a 9 V battery; the receiver is composed of an ultrasound transducer, signal conditioning circuitry, and a digital signal processor (DSP) module connected to a PC via a USB for monitoring purposes. The transmitter and receiver adhere to the external wall of a steel tube 1 m × 270 mm × 2 mm in size and do not require perfect alignment. Flood detection was accomplished innovatively based on the guided wave attenuation characteristic due to energy leakage to water. Two approaches were performed. The first was an off-line signal response based on the entropy analysis of a received pulse using the wavelet transform. Augmentation in signal entropy indicates a flooding member. The second uses real-time digital filtering to increase the signal-to-noise ratio and signal averaging to measure the time–space in-between guided wave pulses. An increase in the time–space of the transmitted pulses indicates a flooding member. Experiments performed in the laboratory successfully identified flooded tubes.

## 2. Leaky Guided Waves

Guided waves are widely used in industry as an effective NDT and SHM tool for the characterization and diagnosis of metal structures, such as plates and pipes. Guided waves can detect discontinuities in ship hulls, submarines, and underwater pipes [14,15,16,17,18], since they can propagate through waveguides immersed in liquids, particularly metals in contact with water [19,20,21,22]. Phase velocity affects guided wave propagation. If the phase velocity is slower than the bulk velocity of the liquid surrounding the structure, its mechanical energy is reflected and mode-converted at the liquid–metal interface, and the wave remains trapped within the waveguide. In this condition, only the material-damping properties dominate their attenuation. However, when the phase velocity is faster than the bulk velocity of the surrounding fluid, its mechanical energy is partially reflected and mode-converted at the interface. The remaining part is refracted and travels into the fluid medium as pressure waves. In this scenario, the out-of-plane wave motion in the structure will transmit into the liquid through the solid–metal interface [23]. The liquid provides a path for the guided wave energy to leak away from the structure, known as leaky guided waves [23], which causes the leaky guided waves to be generally highly attenuated. Besides attenuation, the leaky guided waves present different behaviors in mode shapes and wave speeds compared to the guided waves in free structures [24,25,26,27]. Using analytical methods, several researchers investigated the in-depth dispersion characteristics of immersed metal structures of regular cross-sections, for example, plates, rods, and cylinders [28,29,30,31,32,33,34]. Other researchers employed matrix methods for the dispersion analysis of both embedded and immersed structures, particularly the Transfer Matrix Method (TMM) and the Global Matrix Method (GMM) applied to free, embedded, and immersed waveguides such as plates and cylinders [35,36,37,38,39]. On the other hand, the most common numerical methods for obtaining dispersion curves are the Finite Element Methods (FEMs), Semi-Analytical Finite Element (SAFE) methods, and Boundary Element Methods (BEMs) [40,41,42,43,44,45,46,47,48]. Analytical methods offer good accuracy and require low computational resources; however, they are limited to problems involving simple geometries. Meanwhile, numerical methods require higher computational times but could present a loss of accuracy at high frequencies. In this work, a simple cylindrical structure was considered; hence, the software DISPERSE (version 2.0.20a) [49], which employs GMM, was used to obtain the dispersion curves of a steel tube filled with water.

### Dispersion Curves and Mode Shapes

**Figure 1:**
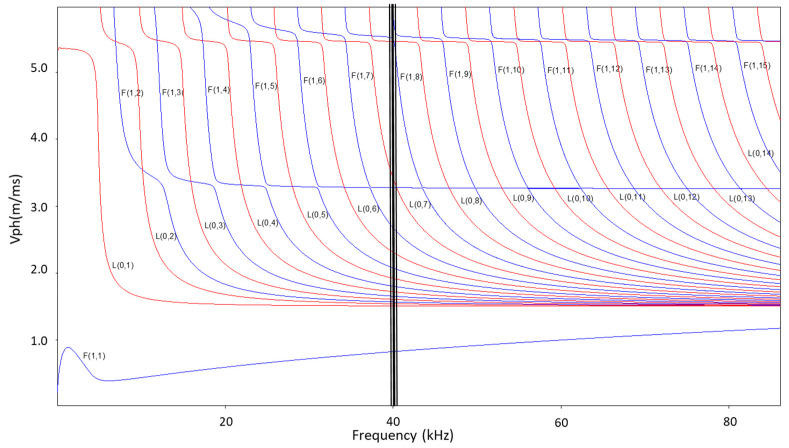
Phase velocity dispersion curve of a steel tube 2 mm × 270 mm diameter mm loaded with water.

There are three principal types of acoustic-guided wave modes, which can propagate along a cylindrical waveguide. These are axis-symmetric longitudinal, *L*(0,*n*), torsional, *T*(0,*n*), and non-axis symmetric flexural, *F*(*M*,*n*), modes [23]. Guided wave modes are characterized by the parameter frequency, phase velocity, and attenuation. Particular attention was paid to the phase velocity and mode shape characteristics of the longitudinal and flexural modes. Velocity dispersion curves were achieved, in terms of a frequency (*f*) and tube thickness (*d*), of a cylindrical waveguide which represent permissible solutions to a frequency equation (*Ω*) of the form:

(1) $$\Omega_{M}\left(a,b,\lambda ,\mu ,f,d,V_{ph}\right)=0$$

where *a* and *b* stand for the inner and outer radii of the cylinder, *λ* and *µ* represent its Lame constants, and *V_ph_* is the phase velocity. The index *M* can be zero or an integer and defines how the fields generated by the guided wave modes vary with the angular coordinate *θ* in a cylinder cross-section. By solving Equation (1) for a known frequency, using the software DISPERSE, the dispersion curves for all three vibrational modes could be obtained. Dispersion curves for a steel tube of 2 mm in thickness and 270 mm in diameter flooded by water are plotted in Figure 1.

**Figure 2:**
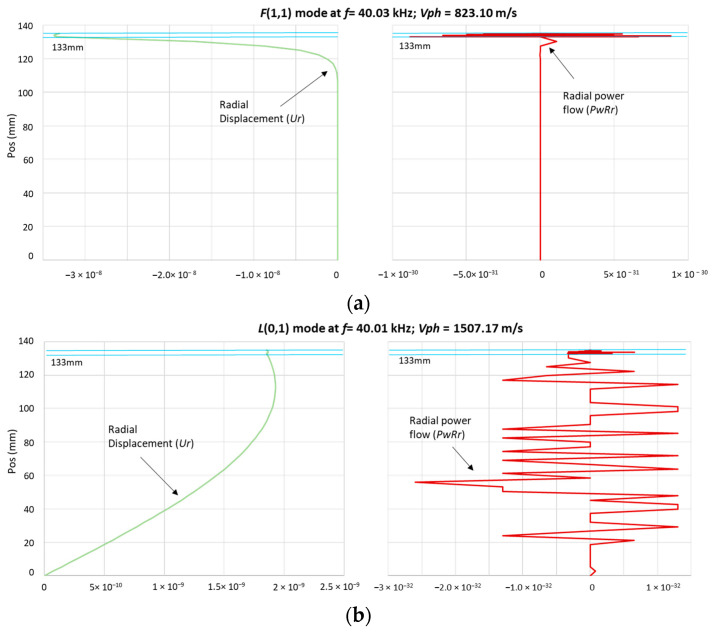
Radial power flow (*P_w_R_r_*) energy and the radial displacement (*U_r_*) at 40 kHz. (**a**) Flexural *F*(1,1) mode; (**b**) longitudinal *L*(0,1) mode.

Generally, in a steel vacuum–pipe–vacuum system at low frequencies, there is only the fundamental *L*(0,1) mode and the flexural *F*(1,1) mode. In a flooded pipe, the profusion of guided wave modes for a given frequency value grows and increases with frequency, as shown in Figure 1. Below 80 kHz, the unique guided wave mode that presents a phase velocity that does not reach the bulk velocity of the water (1500 m/s) is *F*(1,1), and, in theory, non-leakage will occur from steel to water. The rest of the modes present phase velocities above 1500 m/s; hence, energy leakage will arise. Usually, longitudinal axis-symmetric modes are easier to excite than torsional modes, and they are preferred over flexural modes due to the symmetry that allows the inspection through 360° along the circumference of a tube [23]. For longitudinal modes propagating along a tube in contact with a liquid on the outer boundary, the rate of energy transfer per period of oscillation, *P_r_*, is given by:

(2) $$P_{r}=\frac{V_{r}*T_{rr}}{2}$$

where *V_r_* stands for the radial component of the velocity field of the mode, *T_rr_* represents the normal traction component, evaluated at the surface of the tube in contact with the liquid, and the asterisk denotes the complex conjugate [50]. Therefore, the radial component of the exciting modes predominantly influences the amount of leakage and its amplitude loss. In this application, it is desirable to excite guided wave modes that exhibit a certain amount of leakage into the surrounding liquid since this parameter is the basis to determine if there is a flooded member. Taking this into consideration, a frequency excitation of 40 kHz was selected. According to the software DISPERSE mode shapes, the radial power flow (*P_w_R_r_*) energy and the radial displacement (*U_r_*) from the inner wall of the tube to its center abated at 127 mm and 106 mm, respectively, for the *F*(1,1) mode, compared to the *L*(0,1) mode, which continued leaking energy up to its center, as illustrated in Figure 2.

Hence, in a flooded tube, it is expected that the *L*(0,1) mode and excited higher-order modes, Longitudinal and Flexural at 40 kHz, propagate presenting attenuation. Some of these modes will be highly attenuated; meanwhile, others will allow signal detection.

## 3. Wavelet Energy Entropy Signal Processing

A stationary signal is a signal with local statistics that are invariant over its entire duration, where spectral analysis such as fast Fourier transformation (FFT) is commonly employed. Guided waves, however, are dynamic non-stationary signals characterized by frequency dispersion behavior. Traditional frequency-domain analysis methods cannot reveal the characteristics of spectral change over time. Energy spectra depend significantly on the energy variations in different scenarios, for example, attenuations during propagations or changes in the waveform. The wavelet transform (WT) is an effective tool to address the general problem of time–frequency analysis to identify the rapidly varying characteristics of some dispersive wave signals [51,52]. Wavelet analysis is a multi-resolution method used to explore the energy distribution in the time–frequency spectrum. Entropy, on the other hand, characterizes the disorder of distributions. For instance, once the energy of guided waves at a frequency band is attenuated, the amplitude decreases; however, the distribution usually changes scantily. Hence, by using entropy, it is possible to capture the features of the propagation of guided waves. In this work, entropy is combined with the wavelet transform to characterize the energy distributions of guided waves to detect flooding due to energy leakage.

Wavelets are functions defined over a finite interval that hold an average value of zero. The WT decomposes a function into components of different frequencies, which allow one to study each component separately. Hence, the WT represents any arbitrary function as a superposition of a set of wavelets. These wavelets derive from a single prototype wavelet called the mother wavelet, *ψ*(*t*) by dilating or contracting (scaling) the mother wavelet and translating it (shifting). The WT is a mathematics convolution of a signal *x*(*t*) with this mother wavelet. The continuous WT (CWT) form requires an infinite number of operations to obtain the complete reconstruction of a signal, making its results redundant. This redundancy can be overcome by using the discretization version of the mother wavelet, performed for discrete steps in scales and translations via the Discrete Wavelet Transform (DWT), the representation of which can be expressed as:

(3) $$T_{j,k}=\frac{1}{\sqrt{2^{j}}}\int_{-\infty }^{\infty }x(t)\psi (2^{-j}t-k)dt$$

where *T_j_*_,*k*_ are known as wavelet coefficients, *j* is the resolution level, and *k* are each of the elements in the time series. The DWT yields discrete blocks of wavelet coefficients with several elements that reduce as *j* increases [53]. The concept of wavelet frames determines the viability of the wavelet coefficients to represent a full signal. A frame consists of a family of wavelet functions, where the energy of the wavelet coefficients resides within a bounded range of the energy of the original signal [54], given by:

(4) $$AE\leq \sum_{j,k}|T_{j,k}{|}^{2}\leq BE$$

where *E* is the energy of the original signal, *A* and *B* are the frame bounds, and *T_j_*_,*k*_ is the wavelet coefficients. If *A* = *B* = 1, wavelets are orthonormal, and the original signal can be completely reconstructed by:

(5) $$x(t)=\frac{1}{A}\sum_{j,k}T_{j,k}\psi_{j,k}(t)$$

Several basic functions can be used as the mother wavelet, for instance, coiflet 1, Daubechies 1, Daubechies 4, symlet 2, symlet 4, and Gabor. However, the choice of the optimal wavelets includes, besides orthonormality, several criteria such as symmetry, vanishing moments, decomposition level (order), and regularity [51]. The Gabor mother wavelet was selected based on these criteria, which provide the inverse process of reconvening a decomposed signal into its original form. After the Gabor wavelet mother selection and the signal decomposition in wavelet coefficients, a continuous approximation of the signal at each resolution level j can be obtained by:

(6) $$d_{j,k}(t)=\sum_{k=-\infty }^{\infty }T_{j,k}\psi_{j,k}(t)$$

From Equation (6), it is possible to observe the approximation of the signal for each of the *j* resolution levels. For a signal of length *M*, the DWT contains a maximum of *N* stages (*j* = 1, 2, … *N*), with *N* = *log*_2_*M*. The concept of energy is linked with the notions derived from the Fourier theory, where the spectral energy density of a signal *x*(*t*) is *E*(*f*) = |*X*(*f*)|^2^, where *X*(*f*) is the Fourier transform of *x*(*t*). Then, since orthonormal wavelets, by definition, have finite energy, it is possible to measure the energy of each resolution level (*j*) by [55]:

(7) $$E_{j}=\sum_{k}|d_{j,k}(t){|}^{2}$$

Therefore, the total energy for an entire signal is:

(8) $$E_{tot}=\sum_{j}E_{j}$$

By uniting Equations (7) and (8), a degree of occurrence can be assigned equal to the normalized value of the energy to obtain the probability distribution of the wavelet energy for a resolution level as:

(9) $$p_{j}=\frac{E_{j}}{E_{tot}}$$

where *p_j_* denotes the probability distribution of energy at each resolution level *j*, then Σ*p_j_* = 1. Shannon entropy measures the average uncertainty of a random variable. Hence, wavelet energy entropy using *p_j_* is a tool that permits the analysis of the amount of disorder in any distribution [56]. Entropy can be defined by:

(10) $$S=\underset{j}{\sum }p_{j}\ln\left(p_{j}\right)$$

where *p_j_* is the probability distribution of the energy at each resolution level. Since the WT guarantees the accurate decomposition of the original signal into components of different frequencies that allow one to study each component separately, hence, the energy of wavelet coefficients at a particular frequency can be analyzed using the wavelet energy entropy to detect events that modify the energy distribution of the signal.

## 4. FMD-Guided Wave System

**Figure 3:**
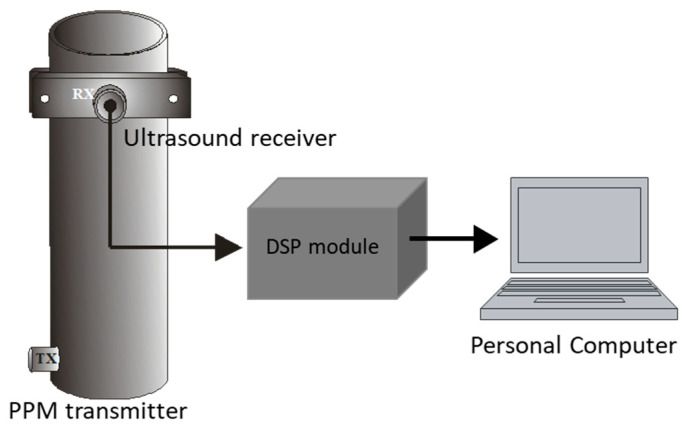
FMD-guided wave PPM system.

**Figure 4:**
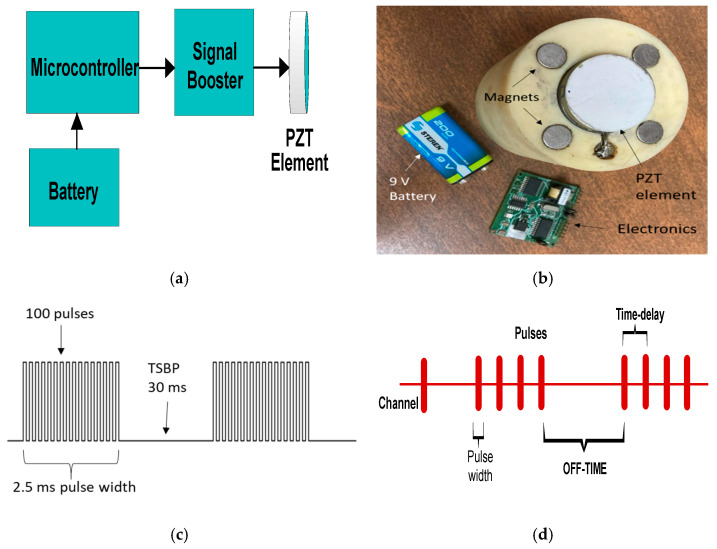
(**a**) PPM transmitter main components; (**b**) actual PPM transmitter design; (**c**) PPM pulse width; (**d**) PPM communication scheme.

An FMD-guided wave system was devised and implemented using a steel tube, a PZT transmitter, and a PZT ultrasound receiver, as illustrated in Figure 3. The transmitter is a stand-alone design that includes a microcontroller, current booster electronics, a single PZT element, and a 9 V battery, as depicted schematically in Figure 4a, and the essential design elements are pictured in Figure 4b. The transmitter uses a modulation-denominated pulse position modulation (PPM), where constant amplitude square pulses of 40 kHz are generated at a predefined time–space between pulses (TSBP). The position of the pulses conveys the signal information. The PPM transmitter was programmed employing the internal flash memory of the microcontroller to generate continuous 100 square pulses of 40 kHz, i.e., 2.5 ms pulse width, and a TSBP set to 30 ms, illustrated in Figure 4c. This process was repeated ten times for signal averaging. An off-time of 150 ms was established as part of the PPM transmitter communication scheme, shown in Figure 4d. The microcontroller digital signal was fed to a current booster and then to a high-frequency step-up pulse transformer with an input/output ratio of 1:8. This signal was applied to a piezoelectric ceramic crystal acquired from APC International, Ltd (Mackeyville, PA, USA).

**Figure 5:**
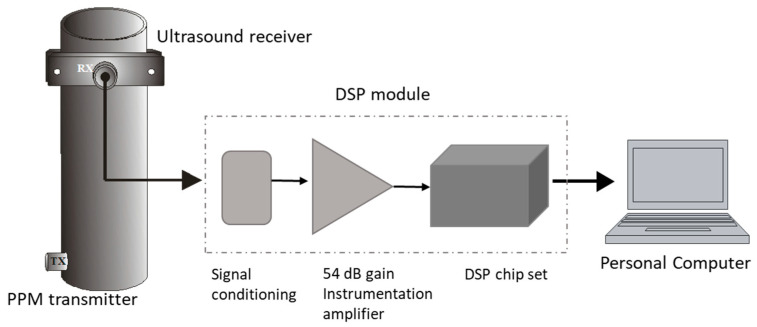
Receiver instrumentation’s main components.

The receiver is composed of a commercial longitudinal ultrasound transducer UTR37KHZ from German Instruments^TM^, a proprietary DSP-based module, which includes signal conditioning, a 54 dB gain instrumentation amplifier, and a USB interface communication to display information in a Personal Computer, shown in Figure 5.

The receiver instrumentation package performs signal enhancement. The DSP module was programmed to execute a sharp bandwidth filter using the standard FIR convolution expression [57].

(11) $$y\left[n\right]=\overset{N-1}{\underset{k=0}{\sum }}h\left[k\right]x\left[n-k\right]$$

where *x*[*n*] and *y*[*n*] correspond to the input and output signals, and the filter impulse response is represented by *h*[*n*]. The real-time digital filter designed to operate with the 40 kHz PPM transmitted information possessed a bandwidth of 4 kHz, the cut-off frequencies of which were 38 kHz and 42 kHz, respectively. Additionally, the DSP module performed signal averaging, *y*_ave_(*k*), where *N* is the number of averages, using the typical expression given by:

(12) $$y_{ave}\left(k\right)=\frac{1}{N}\overset{N}{\underset{n=1}{\sum }}y_{n}\left(k\right)$$

## 5. Experiment Setup

**Figure 6:**
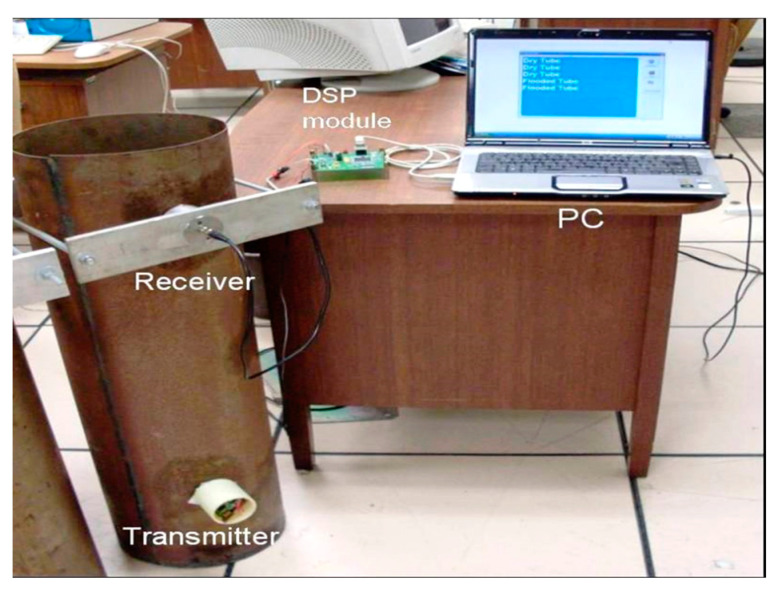
Experiment setup using a hollow steel pipe, 1.5 m × 270 mm × 2 mm, a PPM transmitter, and a DSP instrumentation receiver connected to a PC via USB port.

An experiment was set up in the laboratory using a steel tube 1 m in length, 270 mm in diameter, and 2 mm in thickness, blind at one extreme. A PZT ultrasound transducer, coupled with petroleum jelly, acting as the receiver front-end, was attached with a holder on the top of the pipe and by a coaxial cable connected to the receiver instrumentation. The PPM transmitter was adhered to, without perfect alignment, the opposite extreme at the bottom of the tube using strong magnets. Figure 6 displays this setup.

**Figure 7:**
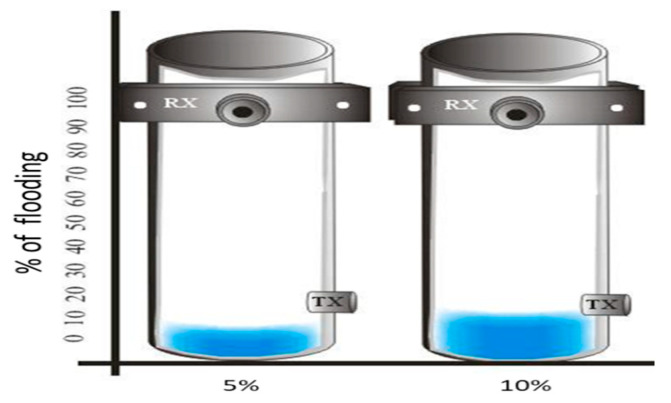
Experiment setup of a tube dry and flooded at 5% and 10% its height.

The FMD-guided wave system was tested in dry conditions, and the tube was filled with water at 5% and 10% of its height, as depicted in Figure 7.

Two approaches for detecting flooding were employed using the same experiment setup of Figure 6. The former analyzes an amplified and FIR-filtered single signal composed of 100 square pulses of 40 kHz off-line, which was saved on a computer for further processing using the wavelet energy entropy tool. The latter uses the real-time analysis of the TSBP by performing digital signal processing on the DSP module.

## 6. Results

### 6.1. Off-Line FMD via Wavelet Energy Entropy

**Figure 8:**
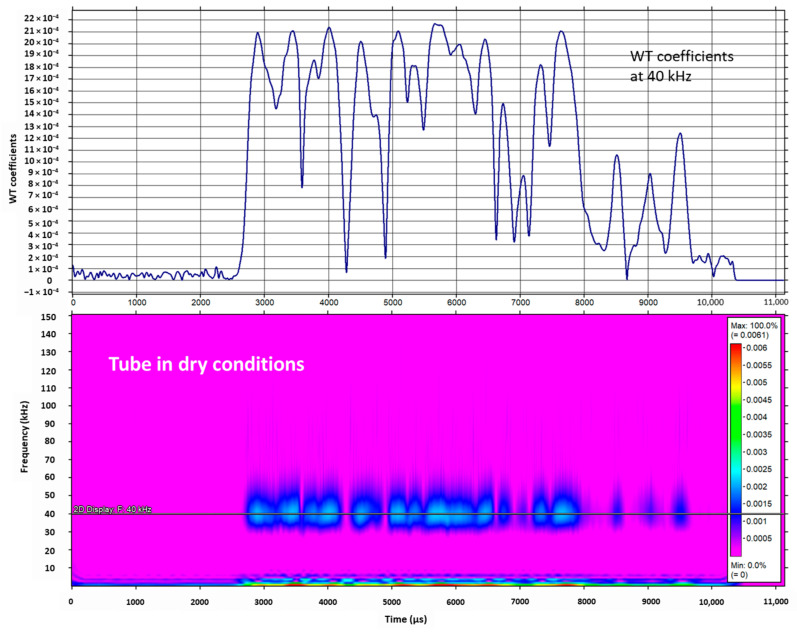

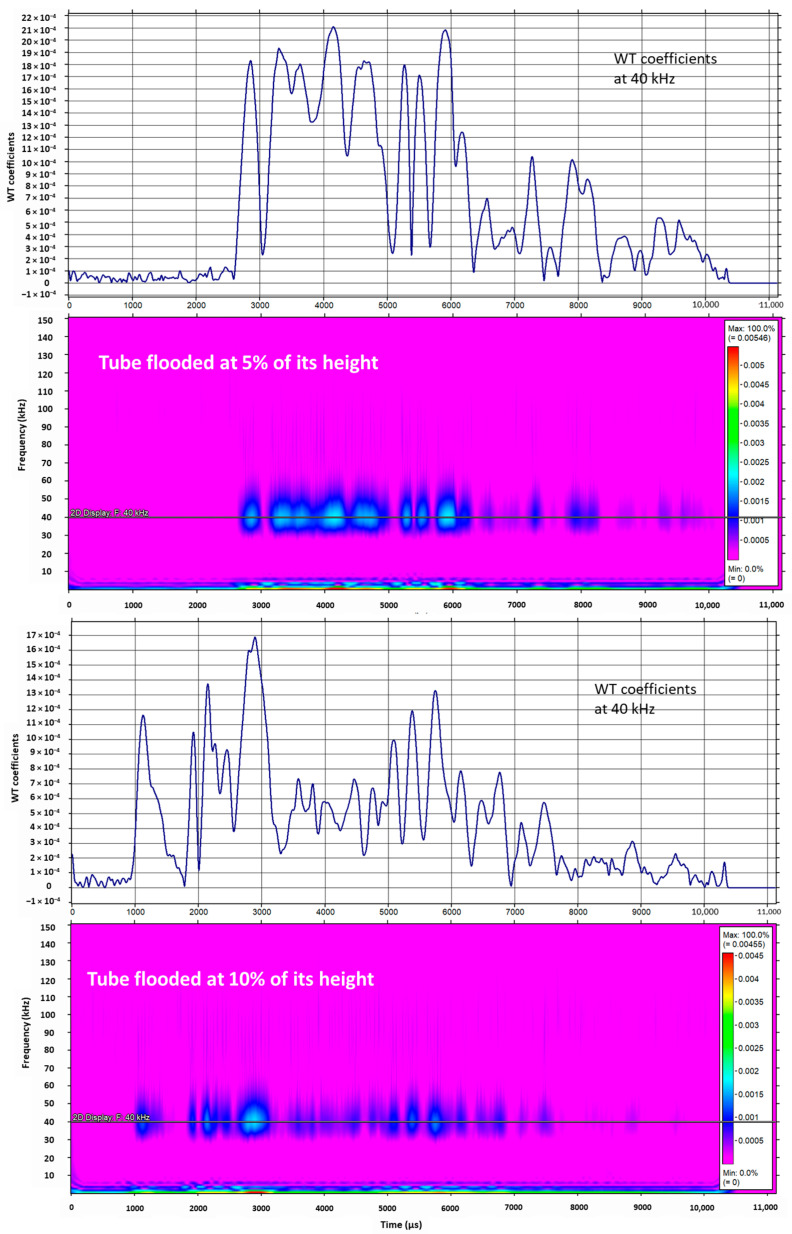
Wavelet coefficients and their spectrogram for a received guided wave pulse centered at 40 kHz using the experiment setup with the tube dry and the tube flooded at 5% and 10% of its height.

Attenuation by guided wave energy leakage from the steel tube to the water is the basis for FMD. The WT using the mother Gabor wavelet was performed using AGU-Vallen© software (R2005.1121) [58] on a received guided wave pulse; it was possible to obtain the wavelet coefficients and a spectrogram at 40 kHz in a range of 0–150 kHz. The signal processing was carried out for signals acquired in a tube in dry conditions and flooded at 5% and 10% of its height, as depicted in Figure 8.

**Figure 9:**
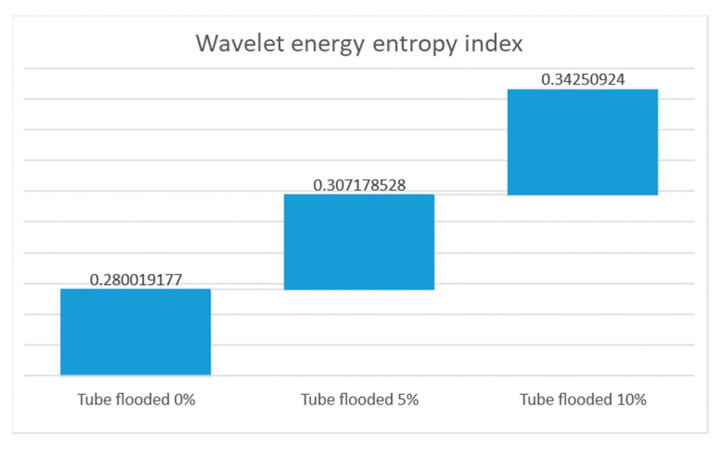
Wavelet energy entropy for the experiment setup in a tube dry and flooded.

The received guided wave energy distribution in Figure 8 shows significant differences for different flooding levels. It exhibits a loss of energy by increasing the percentage of flooding. Once a signal is decomposed in wavelet coefficients via the WT, it is possible to measure the energy of each resolution level (*E_j_*) and the total energy of the signal (*E_tot_*), as indicated in Equations (7) and (8), and obtain the probability distribution of energy at each resolution level (*p_j_*), as shown in Equation (9). Entropy is a quantitative criterion for analyzing probability and the amount of disorder in any distribution; therefore, wavelet energy entropy using *p_j_* displays the amount of disorder of any distribution, as shown in Equation (10), and allows one to detect events that modify the energy distribution of a signal. Figure 9 shows the wavelet energy entropy calculated for the experiment setup for the three conditions of the tube, dry and flooded.

The attained results show an increase in the index of the wavelet energy entropy, which in this application indicates that this signal processing is sensitive for the detection of flooding.

### 6.2. Real-Time FMD via Time–Space between Pulses

**Figure 10:**
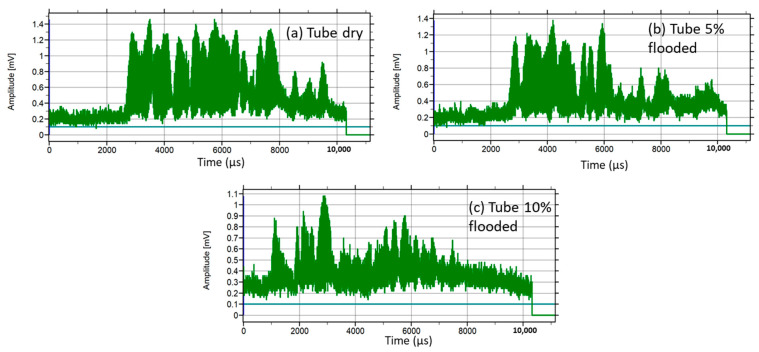
A received guided wave pulse: (**a**) dry tube, (**b**) tube loaded with 5% water, and (**c**) tube loaded with 10% water.

The real-time FMD-guided wave system uses the same elements depicted in the experiment setup of Figure 7. Previously, an author led preliminary work in a steel tube flooded and dry by employing tone pulses of 40 kHz and PPM [59]. In this work, the trial commenced by powering the PPM transmitter. The receiver instrumentation package based on a DSP amplified and filtered out, in real-time, the received guided wave signals, using an instrumentation amplifier, 54 dB gain, and a sharp digital FIR filter 4 kHz bandwidth (38–42 kHz), performed in the DSP module. The DSP carried out an algorithm for the detection of flooding. The fundamental principle of the algorithm considered that guided wave signals, longitudinal and flexural, are sensitive to water loading. Therefore, by using the dry steel tube as a reference, it was expected that the attenuation of the received signals would be minor, as shown in Figure 10a; however, once the tube was water-flooded, the signals exhibited severe attenuation, as depicted in Figure 10b,c.

**Figure 11:**
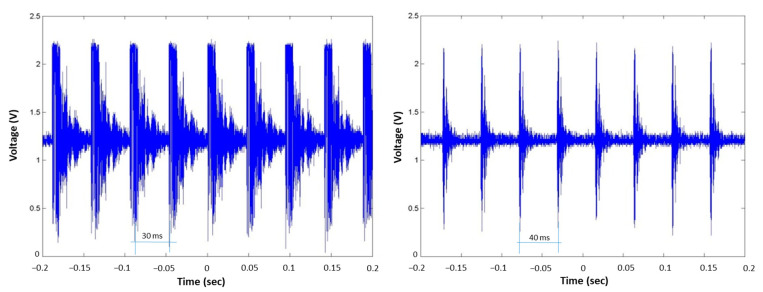
Received guided wave pulses amplified and filtered; (**left**), dry steel tube, TSBP of 30 ms; (**right**), steel tube flooded at 10%, TSBP of 40 ms.

Figure 10 shows that the energy of the received guided wave pulse decreased due to water loading; consequently, the pulse was reduced in amplitude and duration, as shown in Figure 10b,c. The guided waves transmitter generated 10 PPM pulses of fixed 30 ms TSBP. Figure 11a shows a train of acquired pulses in dry conditions, the TSBP of which is taken as a reference. Figure 11b depicts an attenuated train of received pulses when the steel tube was flooded at 10% of its height, which led to a TSBP of approximately 40 ms.

**Figure 12:**
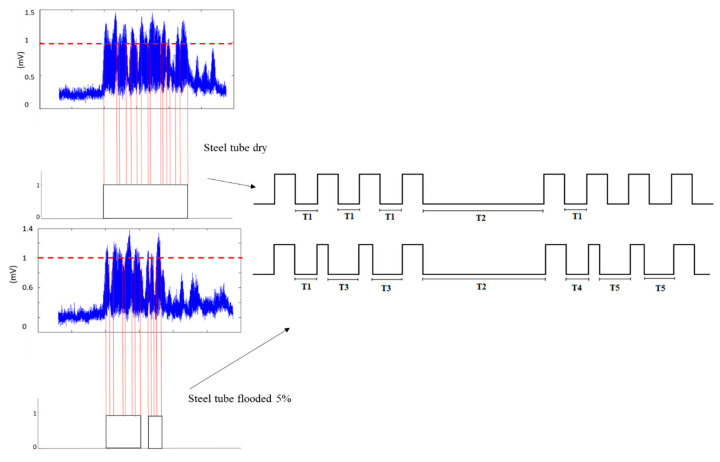
Guided wave analog pulses converted to digital pulses to estimate the TSBP; (**upper**), for a steel tube in dry conditions; (**lower**), for a steel tube flooded at 5% of its height.

The algorithm in the DSP module discriminated between a dry tube and a flooded tube by confirming the number of TSBP, allowing 6 ms of tolerance due to dispersion. Hence, 9-TSBP of 30 ms was expected for every train of 10 pulses. Internally, using a predefined threshold, the DSP converted the guided wave analog pulses into digital pulses to estimate the TSBP, as depicted in Figure 12.

Every time the analog signal went above or below the predefined threshold, the firmware in the DSP generated either a rising or a falling edge; subsequently, the algorithm measured the time elapsed between a falling edge and a rising edge and disregarded measured times shorter than 0.5 ms to yield a logic “1” pulse. Measured times larger than 0.5 ms indicated there was a TSBP. For a steel tube in dry conditions, a predefined 30 ms TSBP illustrated in Figure 12 as T1 was anticipated, and the algorithm computed it as valid. However, if the TSBP was different from T1 due to the leakage of energy in a flooded steel tube, for instance, T3, T4, and T5 depicted in Figure 12, they are considered not valid and they are not computed. T2 is the off-time in-between a train of pulses according to the PPM scheme.

**Figure 13:**
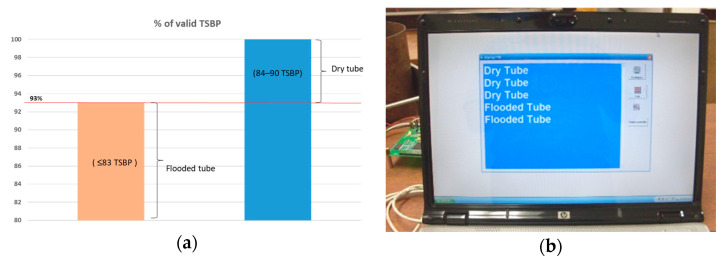
(**a**) Normalized FMD threshold in percentage for the calculated number of TSBP; (**b**) displayed results in a human–machine interface in ASCII standard.

To increase the probability of detection, the number of train pulses incremented to 10, yielding an expected validation of 90 TSBPs. The DSP module computed signal averaging according to Equation (12) of the number of TSBP. The anticipated number of valid TSBPs was compared to the actual number of TSBPs acquired. In the dry condition on the steel tube, the number of valid TSBPs was 88 out of 90. Flooding the steel tube by 5% and 10% of its height increased the TSBP; consequently, it reduced the number of valid TSBPs to 68 and 48 out of 90, respectively. Hence, a threshold for detecting flooding was defined. If the number of TSBP diminished below 84, the tube was considered flooded; otherwise, it was dry. Figure 13a depicts the normalized FMD threshold in percentage for the calculated number of TSBP. The DSP module transmitted continuously in real-time the attained results via a USB in the ASCII standard to a human–machine interface displayed in a PC, shown in Figure 13b. The system successfully detected flooding during laboratory trials.

## 7. Conclusions and Further Work

The present work describes a guided wave system for detecting flooding in a steel tube by exploiting the wave-guide effect of the pipe acting as a communication channel and the transmission and reception of guided wave energy pulses. The system works on the evidence that leaky guided waves, predominantly longitudinal and flexural, exhibit attenuation due to water loading, as yielded by the software Disperse. Two approaches were implemented. The first was an entropy-based methodology that uses the wavelet transform to measure the energy distribution of the reconstructed coefficients of guided wave pulses at 40 kHz. Wavelet energy entropy used to detect changes in energy due to flooding was studied. This methodology showed an increase in the index of the wavelet energy entropy, which in this application indicated the capability of effectively detecting a flood member. The second approach employs the excitation and reception of PPM-guided wave pulses centered at 40 kHz. A proprietary DSP module performs real-time digital signal processing techniques, particularly sharp digital FIR filtering and signal averaging, to measure the TSBP. A reference TSBP was considered for a dry tube using a range of 30 ms to 36 ms, allowing dispersion. A tube filled with water at 5% and 10% of its height led to the attenuation of the excited leaky guided waves that produced an increase in the TSBP, which was computed by a DSP module to successfully identify a flooded tube. Transmitter and receiver PZT probes do not require perfect alignment or frames and do not present any potential danger for users, overcoming traditional ultrasonic and gamma radiation methods. The off-line methodology is successfully performed on a personal computer; however, it exceeds the computational and memory resources of low-complexity embedded systems based on microcontrollers or DSP processors. Additionally, energy consumption is a key parameter in embedded systems, and complex computations involved in the wavelet transform will consume more energy, which will shorten the operational lifetime of a battery-powered stand-alone processor. Nevertheless, this approach opens a line of research that could be employed in future work for correlating the level of flooding with the attenuation of guided wave propagation due to energy leakage. The real-time method is executed on an embedded system based on a DSP processor that performs usual digital signal processing operations and simple calculations to compute TSBP which correctly indicates flooding. This approach does not provide a numerical estimation of a flooded steel tube but offers a pragmatic solution for FMD that can be more suitable for its implementation in the field. These results attained using both approaches are very encouraging, taking the authors to the next stage of this work, which is to design a waterproof guided wave transmitter and receiver to perform trials using a steel tube flooded and immersed in water.

## Data Availability

Not applicable.
